# Ecobat: An online resource to facilitate transparent, evidence‐based interpretation of bat activity data

**DOI:** 10.1002/ece3.3692

**Published:** 2017-12-12

**Authors:** Paul R. Lintott, Sophie Davison, John van Breda, Laura Kubasiewicz, David Dowse, Jonathan Daisley, Emily Haddy, Fiona Mathews

**Affiliations:** ^1^ Hatherly Laboratories Biosciences College of Life and Environmental Sciences University of Exeter Exeter UK; ^2^ University of the West of England Bristol UK; ^3^ BiodiverseIT Dorset UK; ^4^ Mammal Society London UK; ^5^ Heritage Environmental Ltd Perthshire UK

**Keywords:** Chiroptera, conservation tool, data sharing, decision making, ecological consultancy data, environmental impact assessments

## Abstract

Acoustic surveys of bats are one of the techniques most commonly used by ecological practitioners. The results are used in Ecological Impact Assessments to assess the likely impacts of future developments on species that are widely protected in law, and to monitor developments’ postconstruction. However, there is no standardized methodology for analyzing or interpreting these data, which can make the assessment of the ecological value of a site very subjective. Comparisons of sites and projects are therefore difficult for ecologists and decision‐makers, for example, when trying to identify the best location for a new road based on relative bat activity levels along alternative routes. Here, we present a new web‐based, data‐driven tool, Ecobat, which addresses the need for a more robust way of interpreting ecological data. Ecobat offers users an easy, standardized, and objective method for analyzing bat activity data. It allows ecological practitioners to compare bat activity data at regional and national scales and to generate a numerical indicator of the relative importance of a night's worth of bat activity. The tool is free and open‐source; because the underlying algorithms are already developed, it could easily be expanded to new geographical regions and species. Data donation is required to ensure the robustness of the analyses; we use a positive feedback mechanism to encourage ecological practitioners to share data by providing in return high quality, contextualized data analysis, and graphical visualizations for direct use in ecological reports.

## 1 INTRODUCTION

Ecological practitioners collect an enormous quantity of data across a many taxa each year to support planning and conservation decisions. Here, we discuss a new Web‐based, data‐driven tool, Ecobat (www.ecobat.org.uk), that has been developed as both an online data repository and a tool to help ecological practitioners and environmental managers better analyze bat activity data. It provides an objective and standardized output which places activity levels in the context.

## 2 INTERPRETING BAT SURVEY RESULTS

Bat populations can be estimated by counting the number of individuals emerging from summer roosts or within hibernacula; however, roosts can be difficult to find and do not give an indication of the importance of an area for foraging bats. Ecological practitioners therefore frequently use acoustic surveys with static bat detectors to determine species’ presence (e.g., Roche et al., 2011) and to quantify activity levels which can act a surrogate for relative abundance (e.g., Kalko, Villegas, Schmidt, Wegmann, & Meyer, 2008; Lintott, Fuentes‐Montemayor, Goulson, & Park, 2014; and Razgour, Korine, & Saltz, 2011). Acoustic surveys are vital in determining the level of development permitted at a site, or to monitor the effect of a recent development on protected bat species. The use of acoustic monitoring to collect data is relatively cost‐effective; detectors can be automated to run for long time periods and are nonintrusive (Walters et al., 2013), although the process of verifying species records can be time‐consuming and costly. However, the technique is relatively new—static detectors with automated recording systems have only become widely deployed in the last 6 or 7 years—and this technological advance has not yet been matched by standardization of methodologies for analyzing or interpreting these data. This can make the assessment of the ecological value of a site very subjective.

Ideally, an ecological assessment would include the collection of survey data over a large area encompassing both the study site and surrounding landscape, over a meaningful time period, to produce robust results (Zwart, Robson, Rankin, Whittingham, & McGowan, 2015). This level of detail is, however, rarely possible given the economic and time constraints imposed on collecting such a dataset and the difficulties of obtaining publicly available data. Ecological practitioners therefore frequently make judgments and recommendations using a combination of the best available evidence (i.e., survey data) combined with their collective experience and professional opinion (Hill & Arnold, 2012) to determine the importance of a site in a local, regional, or national context.

Recorded bat activity levels are dependent on several factors including species (Vaughan, Jones, & Harris, 1997), seasonality (Russ, Briffa, & Montgomery, 2003), weather (Erickson & West, 2002), and habitat (Lintott et al., 2015). The type of bat detector used also affects detection rates (Adams, Jantzen, Hamilton, & Fenton, 2012). In assessing the relative importance of a site, practitioners must therefore account for how the number of bat passes recorded may have been influenced by these factors. It is therefore likely that an assessment of the ecological value of a site (and the impacts of any proposed development) will vary between practitioners based on level of experience, preferred surveying methodology, and knowledge of the region and/or species (Hulme, 2014).

This lack of consistency creates challenges in making comparisons between sites/projects and in pooling data for further analysis. The use of standardized approaches for data analysis and interpretation allows opportunity to correct for variables such as region, species, and method and facilitates the contextualization of data gathered from an individual site so that decision making is more transparent and defensible.

## 3 OVERVIEW OF APPROACH

The use of acoustic activity data to enable the objective quantification of bat activity has been proposed in North America (Adams, McGuire, Hooton, & Fenton, 2015) and is used to calculate turbine‐specific cut‐in wind speeds for the bat‐friendly operation of turbines in Germany (Behr et al., 2017; Brinkmann, Behr, Niermann, & Reich, 2011); however, to our knowledge, there is no Web‐based tool available which contextualizes bat activity at a landscape scale. The british Mammal Society, in collaboration with the National Biodiversity Network, the Statutory Nature Conservation Bodies (SNCBs), the University of Exeter, and ecological practitioners, therefore designed the Web‐based tool Ecobat. While originally developed for UK users working with bats, it could easily be expanded internationally and modified to accommodate other taxonomic groups.

### 3.1 Data input

**Figure 1:**
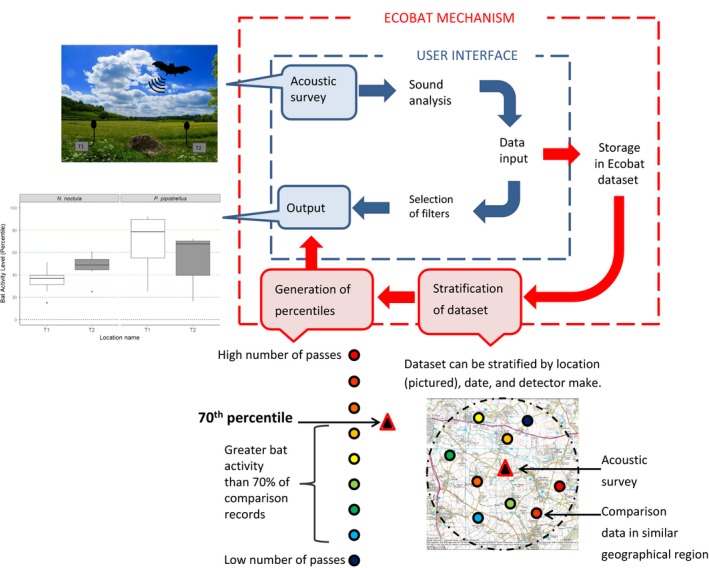
The Ecobat pathway—involving data inputting, processing, and generating an output. Users are asked to specify a number of variables or “filters” (e.g., location, date) to enable stratification of the wider dataset stored in Ecobat

Ecobat is designed to provide environmental practitioners with the ability to split infinitive ‐ corrected deposit bat activity data quicky and securely into a central repository (Table 1; Figure 1). Data can be deposited with varying levels of privacy to accommodate requirements for vulnerable species, sensitive projects, and/or client concerns. Data are currently uploaded via a *pro forma* that is downloadable as a CSV file from the Ecobat website. Each row of data relates to one night of bat activity, per species, per location. Currently, ecological consultants calculate the total number of bat passes recorded across the night and enter this value within the CSV; however, we are developing the capacity to handle raw data directly out of sound analysis software. Uploaded data feed into Ecobat's algorithms, helping to improve the functionality of the site and therefore its utility to environmental practitioners.

**Table 1:** Essential (bold) and nonessential information required when uploading data to Ecobat

Data required	Description
**Location**	**The latitude/longitude or grid reference of the survey site.**
**Sensitivity**	**The confidentiality of the dataset, either locking the data within Ecobat or sharing the dataset with NBN**
**Date**	**The date at sunset**
**Species**	**The species or species group recorded**
**Passes per night**	**Total number of passes per night for each of the species**
**Pass definition**	**The method used to identify a bat pass, for example, a gap of 1 second between calls**
**Detector make and model**	**The manufacturer and model of the bat detector used in the survey**
Detector height	The height of the bat detector used in the survey
Roost proximity	Whether there was a known roost in proximity to the bat detector
Linear features	Whether the bat detector was placed in proximity to any linear features
Anthropogenic features	Whether the bat detector was placed in proximity to any anthropogenic features, for example, buildings and roads
Sunset weather conditions	Temperature, wind speed, and rainfall
Method of sound analysis	Whether automated, manual, or both methods of sound analysis was used
Analysis software used	The software that was used for sound analysis
Detector calibrated	Yes/No—has the detector been calibrated within the past 6 months

### 3.2 Data processing

Ecobat allows users to analyze their data against a comparative reference dataset, for example, records from the same region that were recorded at a comparable time of year (see “Accounting for variability between surveys” below for further details). Percentiles provide a numerical indicator of the relative importance of a night's bat activity. For example, a site that contained bat activity in the 70th percentile would indicate that it had greater activity than 70% of comparison sites (Figure 1). The use of percentiles also enables the level of bat activity to be defined objectively so that there is consistency in the definitions of what is classified as “low,” “moderate,” or “high” activity between ecological assessment statements. We have developed, alongside the UK Statutory Nature Conservation Bodies (SNCBs), the following specifications of activity categories: (1) low activity: 0–20th percentiles, (2) low‐to‐moderate activity: 21st–40th percentiles, (3) moderate activity: 41st–60th percentiles, (4) moderate‐to‐high activity: 61st‐80th percentiles, and (5) high activity: 81st–100th percentiles. These activity categories provide planning authorities and policymakers with the details required to aid making their decision. They are not intended to be prescriptive as, depending on context, different definitions of thresholds may be more appropriate: planning decisions, and the level of mitigation required will depend on a variety of additional factors including the conservation status of the species (e.g., whether it is listed on Annexe II of the EC Habitats Directive) or is considered to be at the edge of its range. However, the use of percentiles and activity categories provides contextualized information about a focal site, facilitating an evidence‐based approach to planning, development, and European Protected Species Licence applications.

### 3.3 Data output

**Figure 2:**
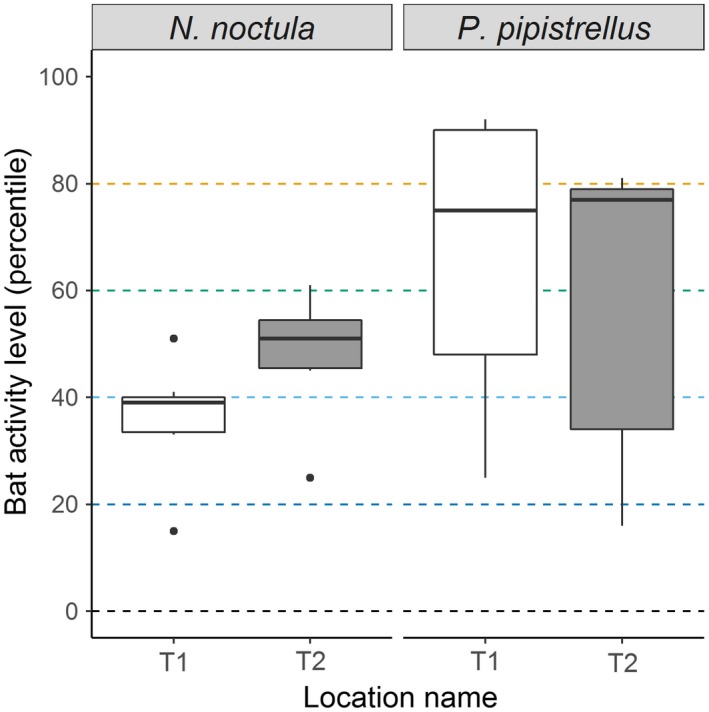
Differences in bat activity between static detectors. The center line indicates the median activity level, whereas the box represents the interquartile range (the spread of the middle 50% of nights of activity). Dashed lines indicate thresholds of bat activity categories (i.e., low activity 0–20th percentiles, low‐to‐moderate activity: 21st–40th percentiles)

**Figure 3:**
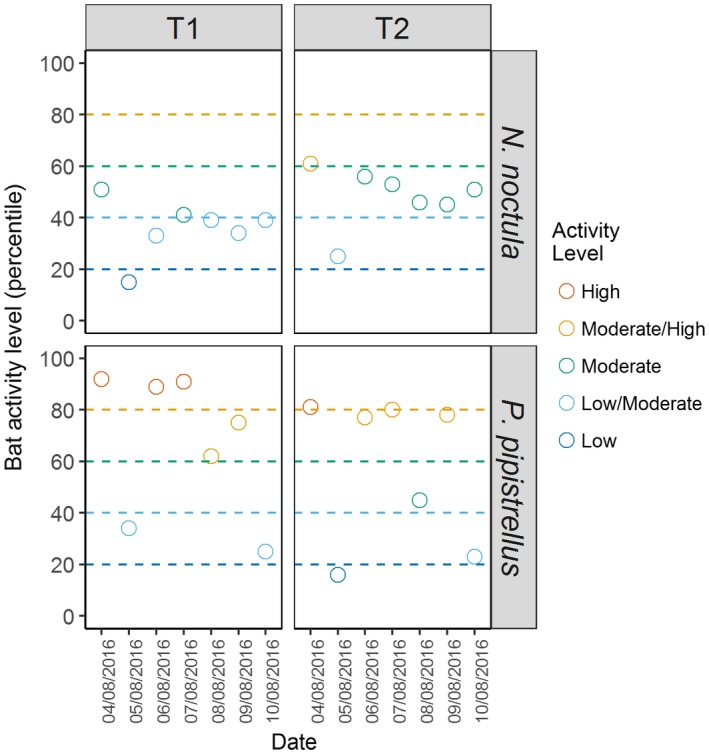
The activity level (percentile) of bats recorded across each night of the bat survey, split by location (here, T1 and T2) and by species

Ecobat provides users who have uploaded data with a downloadable report produced using R Markdown. The report includes: (1) an introductory paragraph that summarises the inputted data; (2) tabulated summaries of key output information (e.g., the maximum and median percentile for each species; Tables 2 and 3); and (3) graphical output. Graphical analyses include a box plot indicating differences in bat activity between static detector locations/sites (Figure 2) and scatterplots showing bat activity level (percentile) against date (Figure 3), temperature, and wind speed (Appendix S1).

**Table 2:** Example output demonstrating how nightly bat activity levels will be assigned to activity categories. Locational data have been abbreviated for brevity

Location (latitude, longitude)	Species/species group	Nights of activity falling into different activity categories				
		High	Moderate/high	Moderate	Low/moderate	Low
50.17, 5.12	*N. noctula*	0	1	5	1	0
50.17, 5.12	*P. pipistrellus*	1	3	1	1	1
50.37, 3.53	*N. noctula*	0	0	2	4	1
50.37, 3.53	*P. pipistrellus*	3	2	0	2	0

**Table 3:** Example output reporting the key metrics recorded for each species across multiple nights of acoustic recording. Reference range size represents the size of the “reference” dataset which the activity data was compared to. Locational data have been abbreviated

Location (latitude, longitude)	Species/species group	Median percentile	95% Confidence intervals	Nights surveyed	Reference range size
50.17, −5.12	*N. noctula*	39	33–41	7	8,120
50.17, −5.12	*P. pipistrellus*	75	34–91	7	12,429
50.73, −3.53	*N. noctula*	51	45–56	7	8,129
50.73, −3.53	*P. pipistrellus*	77	23–80	7	12,238

## 4 TECHNICAL SPECIFICATIONS

Ecobat is developed as an extension of the Indicia toolkit and uses the open‐source content management system Drupal (version 7) to provide a framework for the website. Indicia is an online recording system for wildlife records that can be adapted by organizations to build their own website (Indicia 2017). Example sites which have been built using Indicia include the BBC Breathing Places Ladybird Survey (http://www.bbc.co.uk/breathingplaces/ladybird-survey/), the North East Cetacean Project (http://www.northeastcetaceans.org.uk/), and the Biological Records Centre's iRecord (https://www.brc.ac.uk/irecord/). Custom Indicia code for Ecobat is written in PHP, a widely used, general‐purpose scripting language. All data uploaded by the developers and end users are automatically stored in a PostgreSQL + PostGIS database on the BRC Data Warehouse, hosted by the Natural Environment Research Council's (NERC) Centre for Ecology and Hydrology (CEH). The security of the server is governed by the NERC security policy; servers are backed up nightly, and firewalls are in place to ensure data security and large storage capabilities.

Ecobat uses the open‐source statistical package R (R Core Team 2016), integrated into the Ecobat website using a Shiny App (Chang, Cheng, Allaire, Xie, & McPherson, 2017). This performs analyses and produces output using data retrieved from Indicia's Web services. Shiny Apps allow R to be run from within a website by providing a user‐friendly point‐and‐click interface, while keeping the R code hidden on a server, which can be accessed when the Shiny App is being used. The integration of Shiny with the R package “R Markdown” (Allaire et al., 2017) allows Web users to upload bat activity data and easily generate downloadable, preformatted reports which have been tailored to their dataset.

## 5 ACCOUNTING FOR VARIABILITY BETWEEN SURVEYS

The functionality provided by Ecobat is governed by the trade‐off between accommodating variation in acoustic surveys and providing robust analyses. All percentile outputs therefore contain the “reference range sample size” that a night of activity was contrasted against, to indicate the reliability of the output. As a minimum, we recommend that a reference range dataset is comprised of 200 nights of bat surveying; with smaller datasets, a recommendation to increase the reference range (by expanding sample area or date) is issued in the output. It is also currently necessary to limit the number of variables which can be controlled for, as each stratification subsets the reference dataset, reducing certainty about the assigned percentile. Initially, we have limited the stratification options to variables that we consider essential and are widely considered to exert a strong influence on bat activity; these are:

1. Location—stratify at different geographical scales (100 km^2^, 200 km^2^, UK‐wide);
2. Seasonality—stratify for records within ±30 days of the survey date; and
3. Detector make—stratify results to include only those recorded using the same make of bat detector.

Additionally, we only provide comparisons between records that use the same definition of a bat pass. As the size of the Ecobat database increases, we will be able to allow for the selection of additional variables (Table 1), permitting more nuanced analyses. Similarly, there is a growing trend to use the presence of a bat within a time segment (e.g., 1 minute intervals) as a measure of activity rather than “bat passes” (e.g., Silva, Cabral, Hughes, & Santos, 2017); if this becomes prevalent within ecological consultancy, then this will be incorporated into the Ecobat framework. It should be noted that professional judgment is still required to interpret and frame the results generated from Ecobat within the wider ecological assessment.

## 6 SURVEYING EFFORT

There will be a continual increase in the size of the Ecobat database as survey results are entered. This will improve the robustness of the reference range as the number of data points within any stratified sample will increase. We therefore provide confidence intervals around each of the percentile estimates which indicate the confidence in the output relative to sample size (Table 3); these will become more robust and precise as the database increases. We also provide the sample size of the stratified dataset within each output; this provides consultants and policymakers with a transparent indicator of the reliability of the output. Additionally, long‐term fluctuations in the population size of a species may impact the interpretation of a reference range; for example, the importance of a site for foraging bats may be masked due to a population decline. We will therefore monitor the Bat Conservation Trust's National Bat Monitoring Programme (Barlow et al., 2015) to assess population trends and, if required, implement an option for the end user to stratify the dataset by year (e.g., only include survey data recorded within the previous 3 years within the analysis) to offset this possibility.

## 7 DATA SHARING

Although there has recently been a shift toward open data access within ecological consultancy (e.g., Scottish Windfarm Bird Steering Group, 2015), current data agreements with clients prevent some consultancies from being able to upload data to Ecobat. We therefore suggest there should be an industry move toward sharing ecological data which has the potential to benefit both practitioners and their clients, as it will generate more evidence‐based decisions. Including the requirement to share data in best practice guidelines and new legislation, is likely to encourage a shift in culture toward open‐access data.

Where clients are still reluctant to share data openly, or for sensitive datasets, uploaded records can be classified as “do not publish.” This prevents these records from being publicly accessible; however, they can still be analyzed to produce numerical percentile outputs and they will contribute to Ecobat's underlying algorithms, thus making outputs more robust. Where data are publicly available, they can be shared automatically with the National Biodiversity Network and Local Record Centres, thus streamlining the process and preventing practitioners from having to upload and share data multiple times.

## 8 CONCLUSION AND FUTURE DIRECTIONS

We encourage ecological practitioners to continue to contribute to the project and envisage that Ecobat will become widely used throughout the consultancy and conservation sector. Ecobat works on a positive feedback mechanism, in that the more the data are deposited in the database, the more robust the analyses become; therefore, the more the tool is used, the more useful it will become to its users. As the underlying algorithms have already been developed, there is great potential to expand the tool rapidly to include additional countries and/or taxa.

## CONFLICT OF INTEREST

None declared.

## AUTHOR CONTRIBUTIONS

PRL, FM, DD, and JD conceived the idea; FM gained the funding for the project PRL, SD, JVB, and EH developed the website; PRL, SD, and LK led the writing of the manuscript; all authors contributed critically to the drafts and gave final approval for publication.

## DATA AVAILABILITY

The website for Ecobat can be found within the Ecostat tools of the Mammal Society at http://www.mammal.org.uk/science-research/ecostat/. The source code for both Indicia and Ecobat is publicly available and distributed under the GNU General Public License at https://github.com/Indicia-Team/warehouse and https://github.com/Indicia-Team/ecobat, respectively.
