# Supplementary material for: Ecobat: An online resource to facilitate transparent, evidence‐based interpretation of bat activity data
Source: Ecol Evol. 2017 Dec 12;8(2):935–41. doi: 10.1002/ece3.3692 (PMC5773315; doi:10.1002/ece3.3692)
Supplement: Supplementary file 1 [file ECE3-8-935-s001.pdf]

Appendix 1

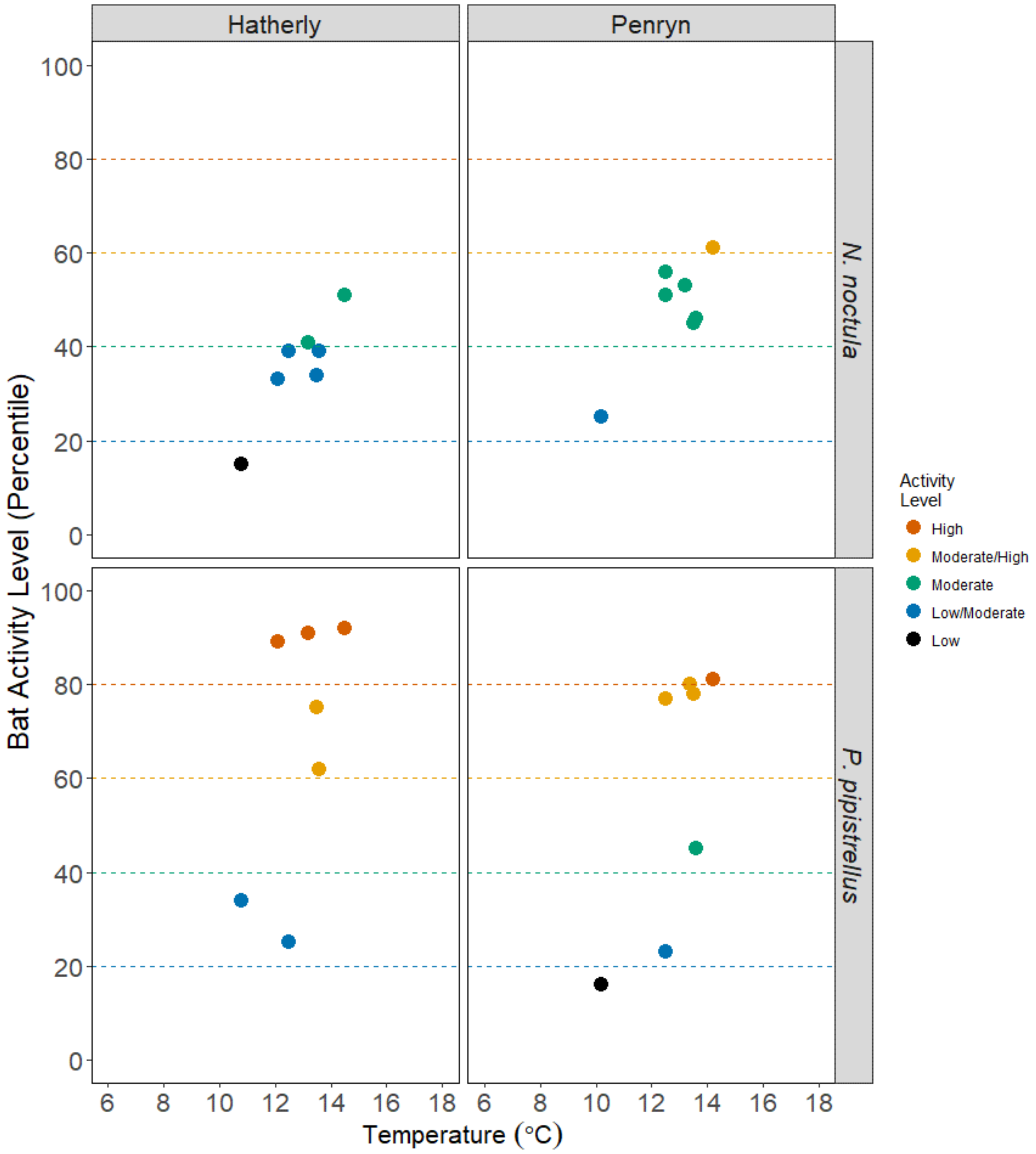

Appendix 1a. The relationship between nightly bat activity and sunset temperature, split by location and species. Dashed lines indicate thresholds of bat activity categories (i.e. low activity 0-20th percentiles, low to moderate activity: 21st-40th percentiles).

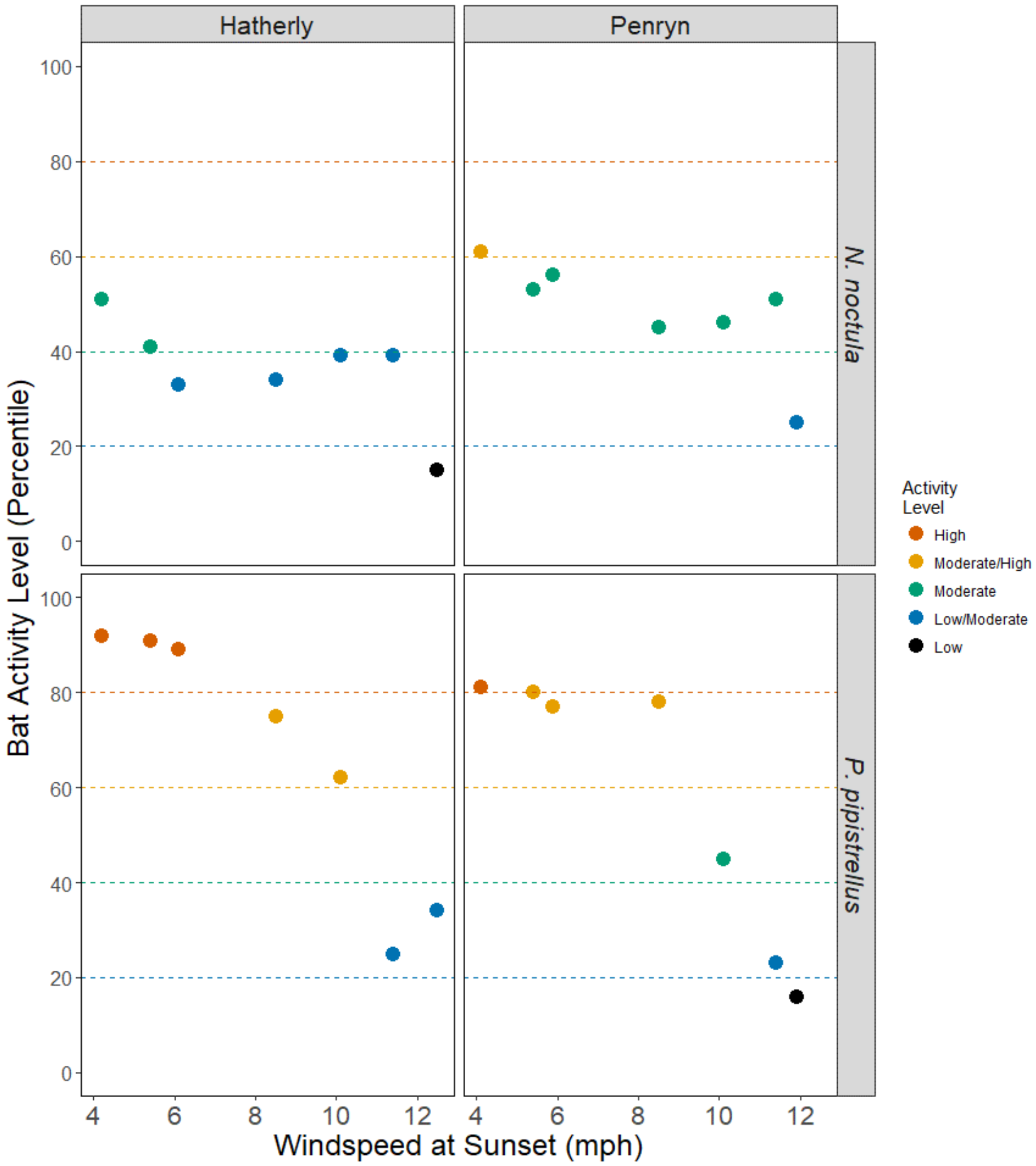

Appendix 1b. The relationship between nightly bat activity and the windspeed at sunset, split by location and species. Dashed lines indicate thresholds of bat activity categories (i.e. low activity 0-20th percentiles, low to moderate activity: 21st-40th percentiles).
